# Indoor radon concentration measurements in dwellings of Riobamba Canton, Central Andes of Ecuador

**DOI:** 10.1093/jrr/rraf074

**Published:** 2026-01-15

**Authors:** Jheny Orbe, Josselyn Guaño, Gabriela Ureña-Callay, Abigail Rivadeneira, Fabian Londo, Nataly Bonilla García, Juan Daniel Padilla Bastidas, Deyaneira Juliana Calle, José Luis Herrera-Robalino

**Affiliations:** Research and Development Group for the Environment and Climatic Change -GIDAC, Escuela Superior Politécnica de Chimborazo (ESPOCH), Riobamba, 060101, Ecuador; Independent researcher in Physics, Riobamba, 060101, Ecuador; Research and Development Group for the Environment and Climatic Change -GIDAC, Escuela Superior Politécnica de Chimborazo (ESPOCH), Riobamba, 060101, Ecuador; Independent researcher in Physics, Riobamba, 060101, Ecuador; Research and Development Group for the Environment and Climatic Change -GIDAC, Escuela Superior Politécnica de Chimborazo (ESPOCH), Riobamba, 060101, Ecuador; Research and Development Group for the Environment and Climatic Change -GIDAC, Escuela Superior Politécnica de Chimborazo (ESPOCH), Riobamba, 060101, Ecuador; Independent researcher in Physics, Riobamba, 060101, Ecuador; Independent researcher, Riobamba, 060101, Ecuador; Independent researcher in Geoscience, Riobamba, 060101, Ecuador

**Keywords:** indoor radon, seasonal variation, Riobamba, Ecuador, Andes

## Abstract

This study aimed to evaluate indoor radon concentrations in Riobamba canton, Ecuador’s central Andean region, and to analyze two factors influencing their variability: surface geology and the age of the dwelling. Radon measurements were conducted in 225 homes using passive monitoring systems, while continuous laboratory monitoring with a Lucas Pylon cell was employed to assess temporal patterns. Concentrations ranged from 9.4 to 152.8 Bq/m^3^, with an arithmetic mean of 49.5 ± 26.6 Bq/m^3^. Ninety-four percent of the homes had radon concentrations below the World Health Organization’s recommended reference level of 100 Bq/m^3^. The calculated average annual effective dose was 1.3 ± 0.7 mSv/year, well below the International Commission on Radiological Protection action level of 10 mSv/year. A statistically significant association was identified between radon levels and the age of the homes; however, no relationship was found between radon levels and the surface geology beneath the buildings. The highest concentrations were observed in houses built before 1925 using traditional techniques such as bahareque, adobe and cancagua. Seasonal analysis revealed minimal variability throughout the year (dry season mean/rainy season mean = 1.04), a result that differs from the well-documented behavior in regions with marked seasonal variability and suggests that seasonal correction factors are not necessary. In addition, a diurnal pattern was evident, which was inversely correlated with indoor temperature and directly correlated with relative humidity. These findings enhance the understanding of radon behavior in the tropical Andean climates characterized by low seasonal variability.

## INTRODUCTION

Radon (^222^Rn) is a chemically inert radioactive gas generated by the decay of uranium-238. It is a component of the air we breathe. Several epidemiological studies have identified a relationship between indoor radon exposure and the incidence of lung cancer, even at relatively low levels, such as those commonly found in homes [1–3].

The soil on which the house is built, the building materials and tap water are the primary sources of indoor radon [4]. The contribution of soil and building materials to indoor radon levels depends on their exhalation rate, which is determined by factors such as radium-226 content, porosity, permeability, water content and ambient conditions [5–7]. On the other hand, radon dissolved in drinking water can be released into the indoor air during activities such as cooking, showering or washing clothes. This source is especially relevant when the water supply comes from underground sources [8, 9].

Radon concentrations in homes vary with the season [10]. These seasonal and diurnal variations are influenced by multiple factors that can differ significantly between countries and regions within a country. Among the most important factors are weather conditions, structural characteristics of the home, ventilation practices, insulation levels and indoor–outdoor air exchange rate [11].

Radon entry rates into homes occur primarily through two mechanisms: convective and diffusive flow [12]. Convective flow is driven by the pressure differential between indoor and outdoor air, caused by the temperature difference between the two. This mechanism facilitates radon entry from the surrounding soil through cracks, foundation openings, construction joints, spaces around pipes and cables and other vulnerable points in the structure. On the other hand, diffusive flow occurs when radon moves from the soil into the indoor air due to the concentration gradients of the gas. This process is slower and depends on the permeability of soil materials. Diffusive flow can be significant in cases where pressure differentials are low or the housing structure is well sealed, limiting convection.

The World Health Organization (WHO) proposes a reference level of 100 Bq/m^3^ to minimize health risks associated with indoor radon exposure. However, if this level is not possible due to country-specific conditions, it is suggested that the reference level not exceed 300 Bq/m^3^ [13]. The European Atomic Energy Community (EURATOM), as outlined in Directive 2013/59/EURATOM, requires member states to establish national reference levels for indoor radon concentrations in both workplaces and residential settings. According to this directive, reference levels for the annual average concentration should not exceed 300 Bq/m^3^, except where national circumstances justify higher values [14].

A comprehensive review of the literature on radon in Ecuadorian homes identifies only two studies. The first, conducted in 2002, involved six Latin American countries (Argentina, Brazil, Ecuador, Mexico, Peru and Venezuela) and determined a national average concentration of 94.3 Bq/m^3^ for Ecuador, based on measurements in 61 homes in Quito, the capital of Ecuador [15]. The second, sponsored by the WHO in 2005 as part of the International Radon Project, culminated in a report published in 2007 that compiled data from 36 member states. The report specifically noted the need to confirm the average indoor radon level of Ecuador [16].

In this context, this paper provides information on indoor radon levels in central Ecuador, as well as an analysis of two factors that influence its variability: the surface geology in which the house was built and its age. The results of radon measurements in 225 homes in 12 towns and parishes in the Riobamba canton during 2024 are presented. The population’s exposure to residential radon was assessed by calculating the annual effective dose associated with radon inhalation. The seasonal and diurnal variation patterns of radon concentrations were analyzed during the year 2023 in a laboratory with the construction characteristics of a typical contemporary home in Riobamba, a constructive style widely used in cities located on the Ecuadorian Andes from 2000 to 3000 m above sea level (m.a.s.l.).

## MATERIALS AND METHODS

### Study area

The Riobamba canton is situated in central Ecuador, at the southern end of the active volcanic zone in the northern Andes. This region differs from the low tropical coastal and Amazonian basins, as well as the insular region of Ecuador, due to its geological context, weather and altitude. The Riobamba canton has an area of 997.9 km^2^ and is located within the province of Chimborazo. The capital of the Riobamba canton, also named Riobamba, is divided into five urban parishes: Lizarzaburu, Maldonado, Velasco, Veloz and Yaruquíes. These parishes comprise the canton’s urban nucleus (hereinafter referred to as the Riobamba urban parish), which concentrates 69.4% (183 318 inhabitants) of the cantonal population. In contrast, the remaining 11 rural parishes, each with its capital town, account for 30.6% (80 730 inhabitants) of the canton’s population [17, 18].

Riobamba city is located 188 kilometers south of Quito. Most of the towns studied are in the inter-Andean depression. However, some are located in the foothills of the Western and Royal Mountain ranges, respectively, at altitudes ranging from 2500 to 3241 m.a.s.l. The reference coordinates for the center of the canton are 1°43′13.55″S and 78°38′42.19″W.

The local geological cover in the parishes studied varies from mainly intermediate to intermediate-felsic Quaternary to Miocene–Pliocene distal volcaniclastic deposits [19, 20], represented, for example, by the geological units of the Cotopaxi, Tarqui and Pisayambo Volcanics; also, locally and to a limited extent toward the East of the study area, Eocene felsic plutonic intrusions are recognized [21], for example, the Pungalá granodiorite intrusion. The basement is metamorphic in the center and east of the canton, being represented by the Guamote terrain, which includes the Guasuntos geological unit consisting of slates and quartzites, and the Alao terrane, which includes the Maguazo, Peltetec and Alao-Paute geological units consisting of turbidite metasediments, meta-andesites, meta-basalts, marbles and cherts [22]. Unlike the basement to the west of the canton, which probably corresponds to the mafic to ultramafic plateau (large igneous province), accreted to the continent in the Late Cretaceous [23].

**Fig. 1:**
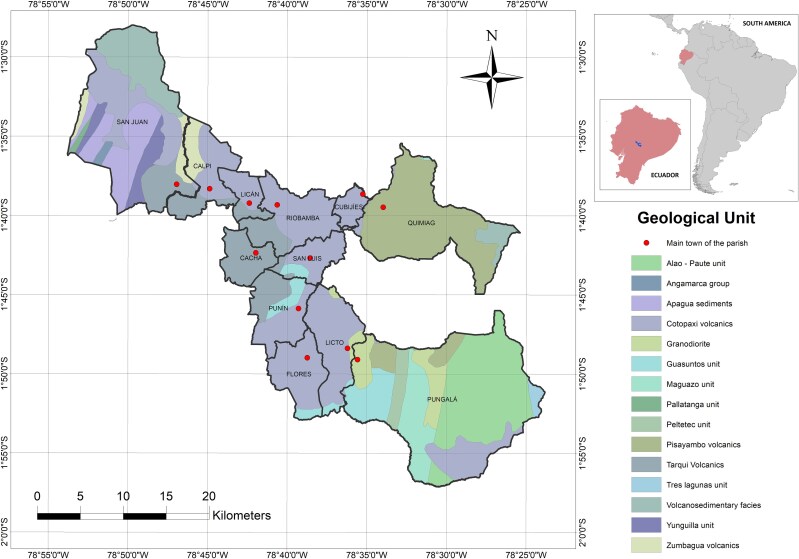
Geological map of Riobamba canton depicting its 12 parishes and their respective capital towns, marked by dots. The upper-right section of the map highlights Ecuador’s position within South America, and the location of Riobamba canton within the country is also depicted.

Figure 1 illustrates the geological map of the study area, showing the locations of the capital towns of the 12 parishes of the Riobamba canton.

Given Ecuador’s location on the equator, there is little seasonality throughout the year. The country experiences two distinct seasons: the rainy and dry seasons. The length of these seasons varies regionally. In the central Andes of Ecuador, where the Riobamba canton is located, the rainy season lasts from October to May and the dry season from June to September [24].

A correlation is observed between the rainy season and higher ambient temperatures, compared to the dry season, characterized by lower temperatures. In 2013, during the dry season, specifically in July, an accumulated precipitation of 6.0 mm and an average temperature of 12.9°C were recorded. In contrast, during the rainy season in May, accumulated precipitation reached 68.3 mm, with an average temperature of 14.4°C. Furthermore, that year the minimum and maximum average temperatures were 6.7 and 22.4°C, respectively [25].

### Indoor radon concentration

Short-term measurements were conducted in all homes using the E-PERM SST detection system (Rad Elec Inc., USA). To determine the consistency of the results, two independent detection systems were simultaneously placed at the monitoring point inside the dwelling. The areas assessed included living rooms, bedrooms and studies. The detectors were installed at a height of 1–2 m above the ground and located at a minimum distance of 1 m from doors and windows. During the measurement period (7 days), the homes were kept in everyday use. It should be noted that the homes have only natural ventilation through windows and no heating systems.

The E-PERM SST detection system consists of an S-model plastic chamber with a volume of 210 ml and a passive detector (Electret Short Term) screwed onto the bottom of the chamber. The chamber includes an on/off mechanism that, when activated, allows radon-laden air to enter and diffuse into its volume through a filtered opening at the top. Alpha particles generated by the decay of radon ionize the air inside the chamber; the resulting negative ions are attracted to the positively charged surface of the electret. This process causes a decrease in the electret’s charge and surface voltage [26]. The potential difference measured before and after exposure enables the calculation of radon concentration in the air. The electret voltage was measured using a voltage reader (SPER-1E Rad Elec).

Radon concentration in dwellings was calculated using Equation (1) [26]:


(1) \begin{align*} {C}_{\mathrm{Rn}}=\left(\frac{\left( Vi- Vf\right)}{(T)\ (CF)}-\mathrm{BG}\right)\times E \end{align*}

where ${C}_{\mathrm{Rn}}$is the radon concentration in $\mathrm{Bq}/{\mathrm{m}}^3$, $Vi\ and\ Vf$are the initial and final electret voltages, respectively, in volts, $T$is the exposure time in days, $\mathrm{CF}$ is a calibration factor for short-term E-PERMS in units of$\kern0.5em V\ por\ Bq/{m}^3\ d,$  $E$ is the correction factor for elevation above sea level and $\mathrm{BG}$ is the radon concentration equivalent to the natural gamma radiation background, which contributes to the electret discharge, in $\mathrm{Bq}/{\mathrm{m}}^3$.

In each measurement, the calibration factor was obtained from the average of the initial and final electret voltage and was calculated using Equation (2) [26]. This equation describes a linear calibration curve determined at sea level, which the electret manufacturer recommends applying for voltages between 700 and 200 V because below 200 V, the curve is not linear.


(2) \begin{equation*} \mathrm{CF}=0.04241+0.0000338\times \frac{\left( Vi+ Vf\right)}{2} \end{equation*}

If radon monitors are used at different elevations, corrections are necessary for more accurate results. For the S chamber, the correction factor becomes significant at elevations above 1200 m.a.s.l. [27].

The elevation correction factor was calculated with Equation (3):


(3) \begin{equation*} E=0.77+6.30\times \frac{h}{\mathrm{30,480}} \end{equation*}

where *h* is the elevation in meters.

The indoor gamma radiation dose rate was measured with the ATOMTEX (AT6102) portable spectrometer. The results ranged from 46 to 78 (nGy/h); these dose rates correspond to radon concentrations ranging from 16 to 26 Bq/m^3^ (BG). The conversion was performed assuming that a background value of 100 nGy/h is equivalent to 32 Bq/m^3^. For other values, the estimate was adjusted using a linear correction [26].

The overall error associated with radon concentration was determined with Equation (4) [26]:


(4) \begin{equation*} {E}_{\mathrm{o}}=\sqrt{{\left(\frac{Vi- Vf}{CF\times T}\right)}^2\left(0.0025+\frac{2}{{\left( Vi- Vf\right)}^2}\right)+{\left(R\times \mathrm{BG}\right)}^2\ } \end{equation*}

This expression considers the error associated with the chamber volume and the thickness of the electrets, the uncertainty related to the electret voltage reading and the relative uncertainty of the natural gamma radiation background (*R*).

The percentage error was calculated with Equation (5)


(5) \begin{equation*} {E}_{\mathrm{percentage}}=100\%\times \frac{E_{\mathrm{o}}}{C_{\mathrm{Rn}}} \end{equation*}

### Statistical analysis

The methodology for evaluating the influence of soil on residential radon concentrations, based on the assumption that soil is the surface layer covering geological formations, consisted of identifying the geological units present within the boundaries of Riobamba canton. The towns studied were assigned to the corresponding geological unit according to their geographic location. The number of residences in each geological unit was subsequently recorded, and the associated radon concentrations were analyzed. Comparison between groups was performed using the non-parametric Kruskal–Wallis test.

The procedure for assessing the impact of housing age on radon concentrations consisted of classifying buildings into three groups. The chronological extremes 1925–1975 were chosen based on historical and construction periods that demonstrate the evolution of construction materials and techniques. The first group consists of houses built before 1925 in the early Republican era (1822–1922), characterized by the use of local materials and traditional techniques such as bahareque (a structure made of reeds or interlaced wood filled with raw mud mixed with straw), adobes (blocks of raw mud mixed with straw, dried in the sun) and cancagua (volcanic soil with physical properties suitable for making blocks-like adobe) [28, 29]. Beginning in the 1960s, Riobamba began a transition from the colonial urban model to modern architectural styles characterized by the incorporation of industrialized materials, such as fired brick and reinforced concrete, combined with traditional methods [30]. It was estimated that 15 years later, in the Riobamba canton, there were two types of houses, those of colonial style and those mixed, built with traditional and industrialized materials. Therefore, the second group includes homes built between 1925 and 1975. Finally, the third group includes buildings built after 1975, which reflect modernization through the widespread use of reinforced concrete, blocks and sealed construction systems [30, 31]. Comparisons between pairs of groups were conducted using the nonparametric Mann–Whitney test to determine whether statistically significant differences existed in radon concentrations.

### Seasonal and diurnal variations

The seasonal and diurnal variations of indoor radon levels were studied, with measurements taken in the Nuclear Techniques Laboratory of the Escuela Superior Politécnica de Chimborazo (ESPOCH), situated at 2818 m.a.s.l. in the city of Riobamba. This laboratory, located on the ground floor, has a structure with stone and concrete foundations, brick walls and a concrete roof. It has five small windows and lacks heating or air conditioning systems. Monitoring was conducted monthly for one week, with 1-hour measurement intervals, using a passive Lucas cell (Model 600P, Pylon Electronics Inc.).

The 600P scintillation cell is an open, airtight metal cylinder with an active volume of 272 ml, which allows a gas sample to diffuse through a polyurethane foam barrier into the cell. It takes ~30 minutes for the cell to reach the same radon concentration level as the environment. The cell’s interior is coated with an alpha-particle-sensitive scintillator of silver-activated zinc sulfide, which emits light pulses when alpha particles strike within an energy range of 4.5–9 MeV. The 600P cell is not a stand-alone system but operates coupled to a monitor, which converts and records the light pulses [32]. For this study, measurements were conducted using the Pylon Model AB7 monitor (hereafter referred to as AB7). The Standard Continuous method was employed, in which the AB7 recorded the total counts at the measurement time (*N*). Net counts (*N*_net_) were calculated as the difference between total counts and background counts (*N*_B_). Radon concentration was calculated manually using Equation (6) [33]:


(6) \begin{equation*} {C}_{\mathrm{Rn}}=\frac{N_{net} PM}{S} \end{equation*}

*C*_Rn_ is the radon concentration in Bq/m^3^, ${N}_{net} PM$ represents the net counts per minute, calculated as the quotient between the net counts and the measurement time in minutes, and S is the cell sensitivity 0.037 cpm/(Bq/m^3^).

Considering that in radioactive counting measurements, the statistical uncertainty is dominated by the Poisson statistics, the percentage error for the radon concentration was calculated using Equation (7)


(7) \begin{equation*} {E}_{\mathrm{percentage}}=100\%\times \sqrt{{\left(\frac{\sigma_{net}}{N_{net}}\right)}^2+{(0.04)}^2\ }\kern0.5em \end{equation*}

(8) \begin{equation*} {\left(\frac{\sigma_{net}}{N_{net}}\right)}^2=\frac{\sqrt{N+{N}_B}}{N_{net}} \end{equation*}

where $\frac{\sigma_{net}}{N_{net}}$ is the relative uncertainty of the net count number, and the value 0.04 is the relative error associated with the cell sensitivity [32].

### Effective dose

The annual effective dose due to indoor radon inhalation was calculated using Equation (9) [34]:


(9) \begin{equation*} D={C}_{\mathrm{Rn}}\times \mathrm{EF}\times O\times \mathrm{DF} \end{equation*}

where *C*_Rn_ is the indoor radon concentration (Bq/m^3^), EF is the equilibrium factor between radon and its daughters (0.4), $O$ is the occupation time in hours (7000 hours) and DF is the dose conversion factor; a value of 9 nSv (Bq h /m^3^)^−1^ was used [35].

## RESULTS

**Fig. 2:**
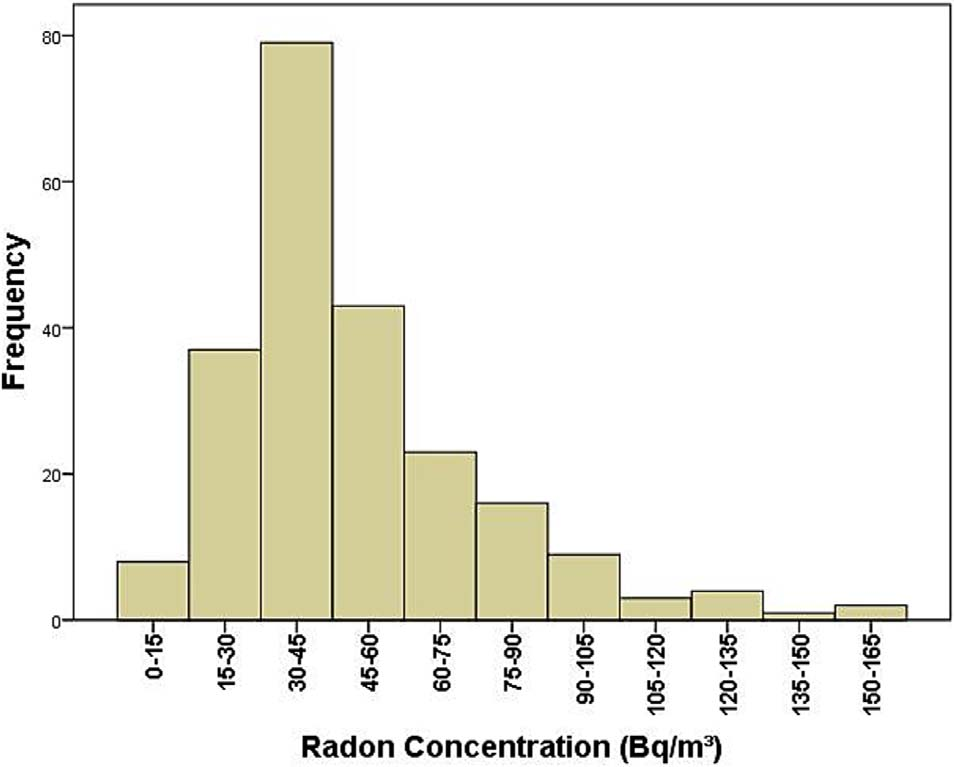
Frequency distribution of radon concentrations in residences within Riobamba canton.

Figure 2 presents the frequency distribution of radon concentrations measured in 225 selected homes in the Riobamba canton. The Kolmogorov–Smirnov test confirmed that the data do not follow a normal distribution (*P*-value = 0.0004).

Radon concentrations in the study area ranged from 9.4 to 152.8 Bq/m^3^, with percentage errors of 40% and 7% respectively. The arithmetic mean concentration was 49.5 ± 26.6 (1σ) Bq/m^3^. The results show that 92% of homes had radon concentrations below 94.3 Bq/m^3^, the national average indoor radon concentration reported for Ecuador in 2002 [15]. Furthermore, only 6% of homes exceeded the reference level of 100 Bq/m^3^ established by the WHO.

Within the Riobamba canton boundaries, 15 geological units were identified (Fig. 1). Nevertheless, the 12 parishes of the canton are situated on only four distinct geological units: Cotopaxi volcanics (U1), Pisayambo volcanics (U2), Granodiorite (U3) and Tarqui volcanics (U4). In the first group were 82% of the houses, 4% in the second, 5% in the third and 9% in the fourth.

Table 1 presents the 12 parishes of the Riobamba canton, along with the number of monitored homes, minimum and maximum radon concentrations (*C*_Rn_), the arithmetic mean (AM) and the standard deviation (SD). The geometric mean (GM), geometric standard deviation (GSD) and the geological units where each town is located are also included. San Luis, Punín and Riobamba parishes recorded the highest mean radon concentrations.

**Table 1:** Descriptive statistics of radon levels in residences of the Riobamba canton and the local geology of the cover

No.	Parish	*n*	Min. C_Rn_ (Bq/m^3^)	Max. C_Rn_ (Bq/m^3^)	AM ± SD (Bq/m^3^)	GM (Bq/m^3^)	GSD (Bq/m^3^)	Geological unit
1	Cacha	8	23.2	46.5	32.3 ± 8.2	31.3	1.3	U4
2	Calpi	13	13.5	66.8	36.8 ± 16.8	32.9	1.6	U1
3	Cubijies	11	13.7	66.3	39.1 ± 12.9	36.6	1.5	U1
4	Flores	7	34.3	49.3	42.0 ± 5.4	41.7	1.1	U1
5	Licán	14	17.3	82.0	46.8 ± 14.7	44.3	1.4	U1
6	Licto	13	18.6	94.8	47.9 ± 22.8	43.4	1.6	U1
7	Pungalá	12	25.1	71.4	44.2 ± 15.0	41.8	1.4	U3
8	Punin	12	28.2	79.2	53.7 ± 14.5	51.7	1.3	U1
9	Quimiag	8	27.6	94.3	46.8 ± 19.1	43.9	1.4	U2
10	Riobamba	83	9.4	140.0	55.8 ± 31.8	46.7	1.9	U1
11	San Juan	13	30.0	58.1	40.1 ± 6.6	39.6	1.2	U4
12	San Luis	31	13.7	152.8	54.9 ± 35.1	46.2	1.8	U1

C_Rn_ = radon concentration; AM = arithmetic mean; SD = standard deviation; GM = geometric mean; GSD = geometric standard deviation; U1 = Cotopaxi volcanics; U2 = Pisayambo volcanics; U3 = Granodiorite; U4 = Tarqui volcanics.

Two important factors that significantly influence indoor radon concentrations were analyzed: the type of geological formation on which the home is situated and its age, as determined through a survey of homeowners. The statistical parameters of radon concentration, grouped by the factors mentioned earlier, are presented in Table 2.

**Table 2:** Statistical parameters of indoor radon concentrations according to the geological formations present in the study area and the age of the homes

Characteristics of the dwelling	*n*	Min. C_Rn_ (Bq/m^3^)	Max. C_Rn_ (Bq/m^3^)	AM ± SD (Bq/m^3^)	GM (Bq/m^3^)	GSD (Bq/m^3^)
Geologic unit						
U1	184	9.4	152.8	51.4 ± 28.3	44.5	1.7
U2	8	27.6	94.3	46.8 ± 19.1	43.9	1.4
U3	12	25.1	71.4	44.2 ± 15.0	41.8	1.4
U4	21	23.2	58.1	37.1 ± 8.2	36.2	1.2
Age of the dwelling						
Before 1925	34	13.7	152.8	74.9 ± 38.1	63.8	1.8
1925–1975	29	18.6	133.3	51.6 ± 26.3	46.2	1.6
After 1975	162	9.4	110.8	43.8 ± 19.3	39.7	1.6
Total dwellings	225	9.4	152.8	49.5 ± 26.6	43.5	1.7

C_Rn_ = radon concentration; AM = arithmetic mean; SD = standard deviation; GM = geometric mean; GSD = geometric standard deviation; U1 = Cotopaxi volcanics; U2 = Pisayambo volcanics; U3 = Granodiorite; U4 = Tarqui volcanics.

**Fig. 3:**
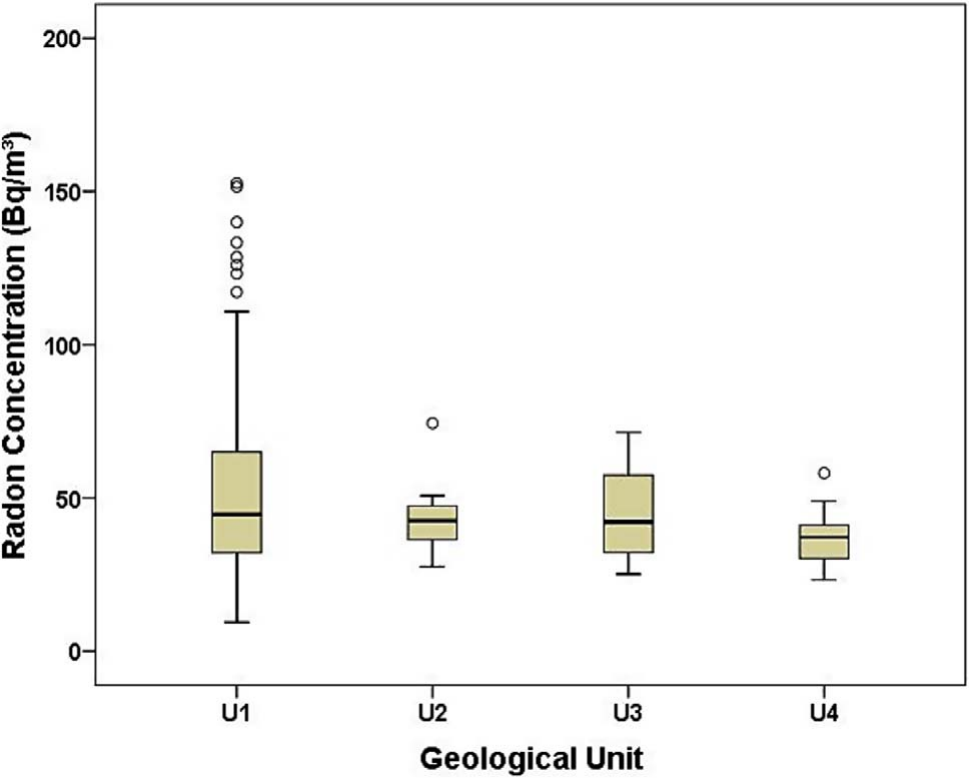
Variability of indoor radon concentrations according to the geological formations in the Riobamba canton. The graph displays the median, as well as the first and third quartiles. Circles represent outlier values. U1 = Cotopaxi Volcanics; U2 = Pisayambo Volcanics; U3 = granodiorite; U4 = Tarqui Volcanics.

The variability of radon levels in homes built on the four geological formations identified in the Riobamba canton is presented in Fig. 3.

The non-parametric Kruskal–Wallis test evidenced no significant differences in indoor radon concentrations between the different geological formations that outcrop in the study area (*P*-value = 0.16).

**Fig. 4:**
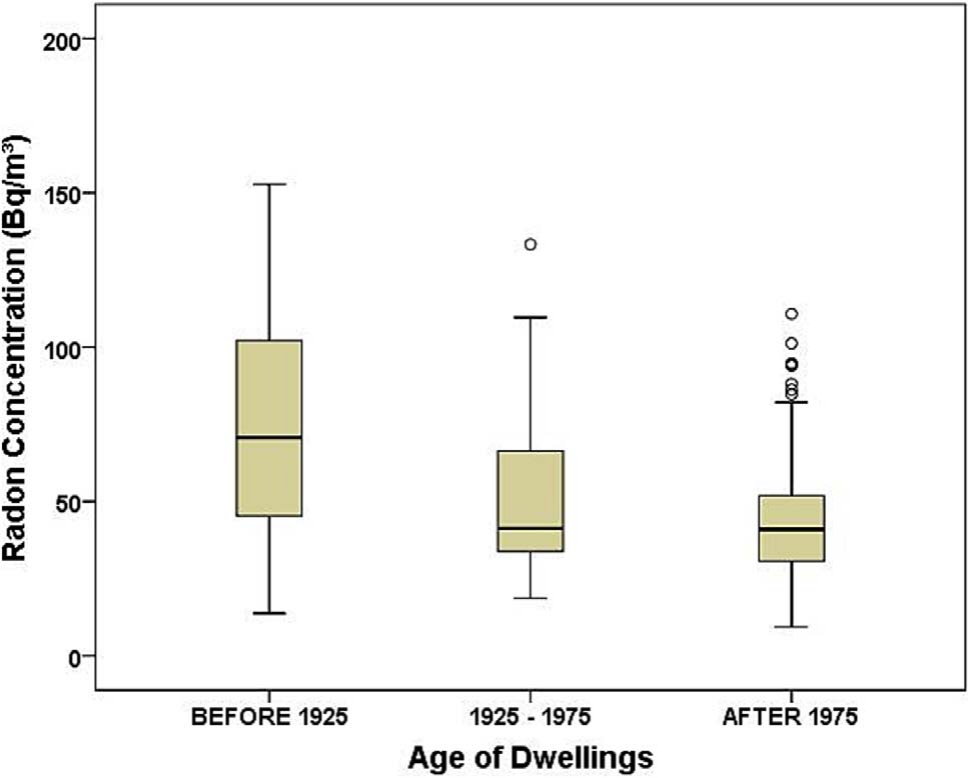
Variability of indoor radon concentrations by home age in the Riobamba canton. The graph displays the median, first and third quartiles and circles represent outlier values.

The dwellings were classified into three groups to evaluate the impact of housing age on radon concentrations: (i) homes built before 1925; (ii) between 1925 and 1975; and (iii) after 1975. In the first group were 15% of the houses, 13% in the second and 72% in the third. Figure 4 shows the variability in radon concentrations as a function of housing age.

The non-parametric Kruskal–Wallis test revealed statistically significant differences in radon levels between at least two groups (*P*-value = 0.00007). Pairwise comparisons were conducted using the non-parametric Mann–Whitney *post hoc* test. A Bonferroni correction was applied to account for multiple comparisons, resulting in an adjusted significance level of α = 0.016. The results showed that radon concentrations in homes built before 1925 were significantly higher than those in homes built after 1975 (*P*-value = 0.0002). The assessment between homes built before 1925 and those built between 1925 and 1975 revealed no statistically significant differences (*P*-value = 0.024). Finally, the comparison between homes built between 1925 and 1975 and those built after 1975 also showed no significant differences (*P*-value = 0.307). These differences can be explained by the structural characteristics of the homes, with emphasis on the construction materials, the architectural design associated with natural ventilation systems and the state of conservation that affects the permeability of the construction.

The annual effective dose due to residential radon exposure for the population of Riobamba canton was estimated using Equation (9). The results indicate that the dose ranges from 0.2 to 3.9 mSv/year, with an average value of 1.3 ± 0.7 mSv/year. According to the International Commission on Radiological Protection (ICRP), remedial actions are recommended when the annual effective dose from radon exposure exceeds 10 mSv/year [36]. In this context, none of the dwellings surveyed reached this action level; therefore, mitigation measures are not deemed necessary.

Table 3 presents the monthly means of minimum, maximum and average ambient temperatures from January to December 2023 in Riobamba city, as well as the monthly radon concentration in the laboratory, calculated from the number of net counts recorded by the AB7 over 168 hours of monitoring, with a measurement time of 1 hour. The absolute error associated with the radon concentration is also shown. Temperature data were collected from the meteorological station situated at ESPOCH.

**Table 3:** Monthly radon concentrations in the laboratory, along with minimum, maximum and average outdoor temperatures in Riobamba city, 2023

Month	Min. Temp. (°C)	Max. Temp. (°C)	Av. Temp. (°C)	Radon concentration (Bq/m^3^)	Absolute error (Bq/m^3^)
January^a^	9.1	18.0	13.4	57.8	2.3
February^a^	9.2	17.5	13.5	59.1	2.4
March^a^	9.4	17.6	13.4	56.4	2.3
April^a^	9.4	17.5	13.5	60.1	2.4
May^a^	9.2	16.8	13.5	58.2	2.4
June^b^	8.3	15.9	12.8	62.2	2.5
July^b^	7.4	15.8	12.5	64.4	2.6
August^b^	7.3	16.2	12.7	56.4	2.3
September^b^	7.8	17.2	13.2	54.1	2.2
October^a^	8.7	17.7	13.2	53.1	2.1
November^a^	8.7	18.3	13.6	52.2	2.1
December^a^	9.2	18.1	13.5	53.3	2.2

^a^Rainy season.

^b^Dry season.

All monthly radon concentrations showed a relative error of 4.1%. It is important to note that only 0.1% corresponds to the statistical uncertainty associated with the number of net counts, while 4% is due to uncertainty in detector sensitivity. This can be explained by the high number of counts recorded during the monitoring week.

**Fig. 5:**
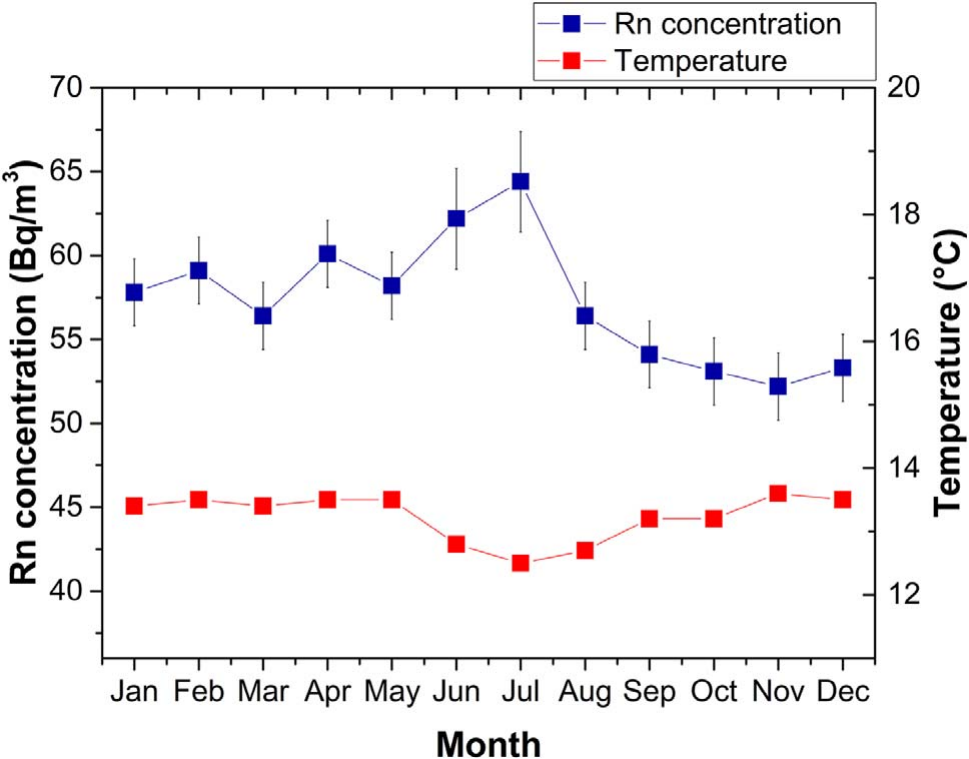
Seasonal variation of radon concentration in the laboratory during the year 2023.

Figure 5 presents the seasonal variation pattern in radon levels, based on monthly data of radon concentration and average temperature presented in Table 3. The highest levels were observed during the dry season, with a mean value of 59 ± 5 (1$\sigma$) Bq/m^3^, coinciding with the lowest mean monthly temperatures of the year and reduced natural ventilation in homes. In contrast, radon levels were slightly lower during the rainy season, averaging 56 ± 3 (1$\sigma$) Bq/m^3^, when temperatures are slightly higher and natural ventilation in homes increases. The mean annual radon concentration was 57 ± 4 (1$\sigma$) Bq/m^3^, while the mean annual temperature reached 13°C. Notably, the highest radon concentration was recorded in July, the coldest month of the year.

The ratio of the mean radon levels during the dry and rainy seasons, measured in the laboratory, was 1.04, indicating a 4% increase in the mean concentration during the dry season compared to the rainy season.

**Fig. 6:**
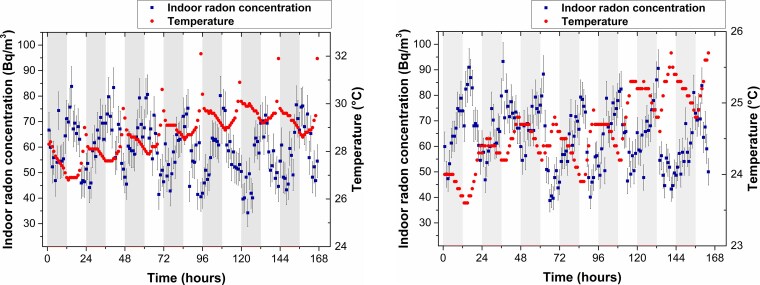
Left: Radon concentrations and temperature inside the laboratory as a function of time during the rainy season (February 2023). Right: Radon levels and temperature during the dry season (July 2023).

Figure 6 presents the radon concentrations recorded inside the laboratory during 2023. On the left side, data are shown during the rainy season, measured hourly from February 10 at 7:00 pm to February 17 at 6:00 pm. During this period, the temperature inside the laboratory ranged from 27°C to 32°C. On the right, radon levels are shown, measured during the dry season from July 9 at 7:00 pm to July 16 at 6:00 pm, with temperatures ranging from 23°C to 25°C. In both cases, a diurnal pattern is evident, as radon levels decrease during the day with rising temperature and increase at night with decreasing temperature. This pattern was observed in all months of the year. In the figures, the shaded areas indicate the nighttime period, between 7:00 p.m. and 7:00 a.m., while the unshaded areas correspond to the daytime period, from 7:00 a.m. to 7:00 p.m.

The pattern of high radon in the morning and low radon in the afternoon is typical of homes where pressure variations between indoor and outdoor air drive radon flux [37]. The diurnal variation pattern can be explained by considering that the laboratory temperature, both day and night, is always higher than the outside temperature, and therefore, convective flow is the predominant mechanism driving radon entry. During the day, the increase in temperature increases the indoor air pressure, causing radon-laden air to be expelled to the outside due to the pressure difference, which reduces the internal concentration. During the night, the decrease in temperature causes a decrease in internal pressure, which favors the entry of radon-enriched outdoor air and, consequently, an increase in concentration.

**Fig. 7:**
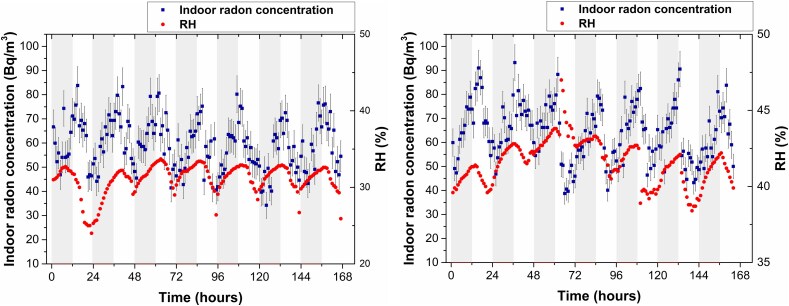
Temporal evolution of radon levels and relative humidity inside the laboratory. Left: Rainy season, February 2023. Right: Dry season, July 2023.

Figure 7 illustrates the radon concentrations recorded in the laboratory during the two monitoring periods described above. The left panel displays data from February 10–17, 2023, with relative humidity ranging from 24% to 34%. The right panel presents data from July 9 to 16, 2023, with relative humidity between 38% and 47%.

In both seasons, radon concentrations were directly correlated with relative humidity. This trend was observed throughout the year.

## DISCUSSION

The results of this study provide valuable insights into the behavior of indoor radon in Riobamba canton, situated in the Central Ecuadorian Andes. Measurements taken in 225 homes revealed concentrations ranging from 9.4 to 152.8 Bq/m^3^, with an arithmetic mean of 49.5 ± 26.6 Bq/m^3^. It is worth noting that 94% of the homes recorded levels below the reference level of 100 Bq/m^3^ recommended by the WHO. Based on these values, an average annual effective dose of 1.3 ± 0.7 mSv/year was estimated for the canton’s population, well below the limit of 10 mSv/year established by the ICRP as a reference level for considering mitigation measures. It is essential to highlight the absence of prior publications in the region that could serve to contextualize or validate the findings presented here.

Although no clear statistical dependence was observed between radon levels and the geological units on which dwellings are based, a relevant correlation with the age of residences was identified. Dwellings built before 1925 with local materials and traditional techniques, such as adobe, cancagua and bahareque, had higher concentrations than those built after 1975, with a new generation of materials: concrete, bricks and blocks. This finding suggests that building materials, ventilation conditions and radon entry pathways into the home play a crucial role in indoor radon accumulation. In older homes, natural ventilation is often limited by the presence of small windows, whereas modern buildings feature large windows that facilitate air exchange. This characteristic favors the accumulation of radon in old dwellings. On the other hand, radon can also enter older homes more easily than modern ones due to their construction characteristics and the natural deterioration that occurs over time. Traditional materials, due to their porous nature, allow gases to enter, while fissures and cracks in floors and walls, caused by earthquakes or ground settlements, act as direct infiltration channels. Additionally, older homes often lack sealed foundations or impermeable barriers.

Seasonal analysis of radon concentrations in the laboratory showed minimal variation throughout the year. Higher concentrations were recorded during the dry season, when temperatures are lower and natural ventilation is reduced, and slightly lower values ​​were recorded during the rainy season, characterized by a slight increase in temperature and natural ventilation. The ratio between the two seasons was 1.04, representing a 4% increase in the dry season. This behavior contrasts with findings from countries characterized by well-defined seasons, cold winters and warm summers, where indoor radon concentrations have been shown to vary seasonally, typically increasing during winter and decreasing during summer [38–40]. In Italy, for example, a representative survey carried out between 1989 and 1998 in 5631 dwellings in all 21 Italian regions reported that regional values of the geometric mean winter/summer ratios ranged from 0.81 to 2.58, with a national geometric mean of 1.23 [41], implying a 23% increase in winter. In Finland, a study of 3074 homes revealed that the mean winter/summer concentration ratio in low-rise residential buildings was 1.28, while in dwellings with winter concentrations of <50, 50–100, 100–200 and >200 Bq/m^3^, the ratios were 0.97, 1.22, 1.34 and 1.55, respectively, resulting in an increase of 22%, 34% and 55% in winter [42]. In summary, the low seasonal variation in radon concentrations in Riobamba reflects the canton’s low climatic seasonality, which is caused by its location near the equatorial line, where solar radiation is relatively constant throughout the year, and its high altitude, which moderates temperatures, resulting in a temperate climate with limited annual temperature variations. Although there are rainy and dry seasons that affect precipitation, the temperature varies little, with a marked daily temperature range (mild days and cold nights).

In climates with strong seasonality, these patterns justify the application of correction factors to estimate annual concentrations from short-term measurements [43–45]. For example, in southwest England, these factors are significant and could alter the estimated annual average by up to 35% for a 6-month measurement and up to 56% for 3-month periods [46]. However, the limited seasonal variation observed in radon levels in Riobamba does not justify the application of these factors. This finding could be generalized to other cities in Ecuador’s Andean region and even extended to tropical Andean environments in general, which share similar climatic characteristics.

Finally, continuous laboratory monitoring using a Lucas cell (Pylon) enabled the identification of a clear diurnal pattern in radon concentrations. These levels decreased during the day as indoor temperature increased and rose at night when the temperature dropped. This inverse behavior, characterized by maximum levels in the morning and minimum levels in the afternoon, can be explained by hypothesizing that convective flow is the predominant mechanism of radon entry. This flow is generated by the pressure gradient between the indoor and outdoor air, caused by the temperature difference between the two environments. The laboratory temperature remains above the outdoor temperature both during the day and at night. Additionally, radon concentrations showed a pattern consistent with relative humidity, possibly due to the influence of standard variables such as temperature and ventilation.

In conclusion, in the Riobamba canton, only 6% of dwellings had radon levels above the reference value established by the WHO. Annual inhalation doses do not pose a health risk to the population. A statistically significant correlation was found between radon concentrations and the age of dwellings, while no relationship was observed between radon concentrations and the type of geological substrate. Seasonal variation was minimal (4%), ruling out the need for correction factors. Furthermore, a diurnal pattern associated with temperature and humidity was observed.
